# The NIA Interventions Testing Program Announces the 2016 Solicitation of Proposals

**DOI:** 10.1111/acel.12419

**Published:** 2015-11-11

The National Institute on Aging (NIA) Interventions Testing Program (ITP) investigates dietary supplements purported to extend lifespan and/or delay the onset of disease and disability. The NIA ITP tests such compounds in mice, using a variety of measured endpoints to assess the efficacy of interventions. The NIA ITP is not a mechanism for funding sponsors’ laboratories to perform the work, but rather it is a collaborative effort between the three NIA‐funded testing sites and the sponsors who propose interventions for study. The sponsor's role is to provide the rationale for investigating the intervention, make recommendations on the dose, route and timing for administration of the intervention, and propose assays and measurements to document the efficacy of the intervention. The sponsors have access to all data developed from the treated mice, assist in analysis of the data and co‐author on resulting publications.

The ITP provides a venue for early exploratory studies on compounds with potential to impact aging and lifespan and also an opportunity to follow up on significant findings. One of the first exciting findings from the ITP was the report that treatment with rapamycin, an inhibitor of the mTOR kinase, produced robust lifespan in both male and female mice, albeit skewed in favor of females (Harrison *et al*., 2009). The ITP followed up with a more in depth study, including a dose–response curve for the lifespan analysis and further characterization of the cellular effects of rapamycin treatment. At the two lowest doses, 4.7 and 14 ppm in the food, female mice again responded with a greater extension of lifespan than did male mice, while at the highest dose, 42 ppm, males and females showed similar increases in median lifespan, 23% and 26%, respectively (Miller *et al*., 2014). There was a sex difference in the blood levels of rapamycin, but that did not explain the difference in lifespan, as the greatest difference in the blood levels was at the highest dose, where the effect on lifespan was very similar for males and females. And while rapamycin and dietary restriction both impact the mTOR pathway, this study demonstrated distinct difference in some metabolic parameters between rapamycin and dietary restriction, such as in the effect on leptin and FGF‐21 levels (Miller *et al*., 2014).

The NIA ITP is soliciting proposals for compounds to enter the study in 2017. The deadline for receipt of proposals is February 24, 2016. Information on the NIA ITP and guidelines for proposal development are posted at: http://www.nia.nih.gov/research/dab/interventions-testing-program-itp. Questions should be directed to Dr. Nancy Nadon (nadonn@nia.nih.gov).
